# IGF2BP2: an m^6^A reader that affects cellular function and disease progression

**DOI:** 10.1186/s11658-025-00723-9

**Published:** 2025-04-09

**Authors:** Siyi Liu, Shan Liao, Junyu He, Yanhong Zhou, Qian He

**Affiliations:** 1 Department of Radiation Oncology, The Affiliated Cancer Hospital of Xiangya School of Medicine Central South University/Hunan Cancer Hospital, Changsha, 410013 Hunan China; 2 Cancer Research Institute, Basic School of Medicine, Central South University, Changsha, 410011 Hunan China; 3 Department of Pathology, The Third Xiangya Hospital of Central South University, Changsha, 410013 Hunan China; 4 Department of Clinical Laboratory, Brain Hospital of Hunan Province (The Second People’s Hospital of Hunan Province), Changsha, 410007 Hunan People’s Republic of China

**Keywords:** IGF2BP2, Ferroptosis, Epithelial–mesenchymal transition, Cell stemness, Angiogenesis

## Abstract

Insulin-like growth factor 2 messenger RNA (mRNA)-binding protein 2 (IGF2BP2) is a widely studied N^6^-methyladenosine (m^6^A) modification reader, primarily functioning to recognize and bind to m^6^A modification sites on the mRNA of downstream target genes, thereby enhancing their stability. Previous studies have suggested that the IGF2BP2-m^6^A modification plays an essential role in cellular functions and the progression of various diseases. In this review, we focus on summarizing the molecular mechanisms by which IGF2BP2 enhances the mRNA stability of downstream target genes through m^6^A modification, thereby regulating cell ferroptosis, epithelial–mesenchymal transition (EMT), stemness, angiogenesis, inflammatory responses, and lipid metabolism, ultimately affecting disease progression. Additionally, we update the related research progress on IGF2BP2. This article aims to elucidate the effects of IGF2BP2 on cell ferroptosis, EMT, stemness, angiogenesis, inflammatory responses, and lipid metabolism, providing a new perspective for a comprehensive understanding of the relationship between IGF2BP2 and cell functions such as ferroptosis and EMT, as well as the potential for targeted IGF2BP2 therapy for tumors and other diseases.

## Introduction

m^6^A is one of the most common reversible modification forms of eukaryotic messenger RNA (mRNA) [1]. As a key epigenetic mechanism, m^6^A modification directly controls RNA metabolic processes, including mRNA processing, mRNA export, translation initiation, mRNA stability, and lncRNA (long noncoding RNA) biogenesis, thereby regulating various processes from cell differentiation and apoptosis to treatment resistance, immune response, and ultimately cell function [2, 3].

There are three types of proteins involved in the biological process of m^6^A modification: m^6^A methyltransferases (the “writers”), m^6^A demethylases (the “erasers”), and m^6^A recognition factors (the “readers”) [4]. The m^6^A writer, composed of the catalytic subunit MAC and the regulatory subunit MACOM, is primarily responsible for m^6^A deposition on specific substrate RNAs [5]. The deposition of m^6^A on RNA can alter the secondary structure of local RNA, creating one or more binding sites for m^6^A effectors, thereby affecting the interaction between RNA and RNA-binding proteins (RBPs) [6, 7]. m^6^A modification mediates alternative splicing by influencing the binding of small nuclear RNAs (snRNAs), key components of spliceosomes, or splicing factors to pre-mRNAs [8–10]. m^6^A erasers, such as fat mass and obesity-associated protein (FTO) and alkB homolog 5 (ALKBH5), can effectively mediate the removal of RNA m^6^A modifications and maintain the reversibility of the m^6^A regulatory system [11]. The m^6^A readers are RBPs that recognize and selectively bind to m^6^A sites (with the exception of YTHDC2), guiding the fate of target RNAs [12, 13]. These three types of proteins play an irreplaceable role in the m^6^A modification process.

The insulin-like growth factor 2 mRNA-binding protein family (IGF2BPs) is a recently identified class of RBP with m^6^A reader function [14]. As m^6^A-binding proteins, IGF2BPs possess six characteristic RNA-binding domains, including two RNA recognition motifs (RRM1 and RRM2) and four K-homology (KH) domains (KH1 to KH4) [15]. IGF2BPs preferentially bind to the consensus sequence of “UGGAC” (containing the m^6^A core motif of “GGAC”) [16]. Most IGF2BP binding sites (92%) are located in protein-coding transcripts (mRNA) and are highly enriched in the 3′-untranslated region (3'-UTR) and near stop codons [16]. Additionally, the KH3-4 di-domain is essential for m^6^A recognition and binding, while KH1-2 may play an auxiliary role. Meanwhile, posttranslational modifications of the regions flanking KH3–4 may also be important for the selectivity of IGF2BPs [15, 17].

IGF2BP2 is a member of the IGF2BP family [3]. Huang et al. found that high-confidence targets of IGF2BP2 often have longer half-lives than their nontarget counterparts and play a crucial role in enhancing the stability of target mRNA [16]. During the process of exerting its mRNA-stabilizing function, IGF2BP2 relies on RNA stabilizers such as ELAV-like RNA-binding protein 1 (HuR) and matrin 3 (MATR3) [18, 19]. Specifically, IGF2BP2 can recruit HuR to protect m^6^A-containing mRNA from degradation and promote its translation [16]. Additionally, during heat shock and recovery, IGF2BP2 colocalizes with stress granules and shuttles between ribosomal and nonribosomal components, revealing its role in mRNA stability and storage under stress conditions [16]. Numerous studies have shown that IGF2BP2 is involved in the development and progression of various biological processes and malignant tumors, such as cancer metabolism, cell apoptosis, invasion, and metastasis, by regulating different types of RNA in various cell types and pathways.

Today, m^6^A modification is a subject of widespread interest. An increasing number of researchers recognize that the interactions of IGF2BP2, as an m^6^A reader, with m^6^A writers or erasers play a significant role in the progression of human metabolic disease and tumors. For example, Wang et al. primarily reviewed the role of IGF2BP2 in metabolic diseases such as diabetes, nonalcoholic steatohepatitis, obesity, and fatty liver. They also elucidated the mechanisms by which IGF2BP2 regulates breast, pancreatic, and esophageal cancers through miRNA and lncRNA [20]. However, with further research into cellular functions such as ferroptosis, angiogenesis, and cellular inflammation, IGF2BP2 has been found to play an important role in these processes as well. In this article, we focus on summarizing the specific molecular mechanisms by which IGF2BP2 affects ferroptosis, epithelial–mesenchymal transition (EMT), stemness, angiogenesis, inflammatory responses, and lipid metabolism. We also update recent research on the impact of IGF2BP2 on disease prognosis and treatment, providing a theoretical basis for a comprehensive understanding of IGF2BP2-m^6^A modifications.

## IGF2BP2 affects cell ferroptosis

Ferroptosis, an iron-dependent cell death program, is characterized by redox imbalance and subsequent toxic lipid peroxidation, showing great potential in cancer therapy [21, 22]. Biochemically, cells undergoing ferroptosis exhibit decreased levels of glutathione (GSH) and glutathione peroxidase 4 (GPX4), as well as an accumulation of lipid peroxidation [23]. In the past 3 years, a multitude of studies have found that, in tumor cells such as esophageal squamous cell carcinoma (ESCC) and hypopharyngeal squamous cell carcinoma (HPSCC), as well as in normal cells such as human colon epithelial cells, alveolar epithelial cells, and primary microvascular endothelial cells, IGF2BP2 can regulate the stability of ferroptosis-related proteins such as GPX4, solute carrier family 7, member 11 (SLC7A11), and hypoxia-inducible factor (HIF-1) alpha mRNA stability, or accelerate the degradation of downstream target gene activating transcription factor 3 (ATF3) in an m^6^A modification-dependent manner, thereby affecting ferroptosis and playing a more critical role in disease progression [24–29].

### IGF2BP2 enhances mRNA stability of downstream target genes and inhibits cell ferroptosis through an m^6^A modification-dependent mechanism

**Fig. 1:**
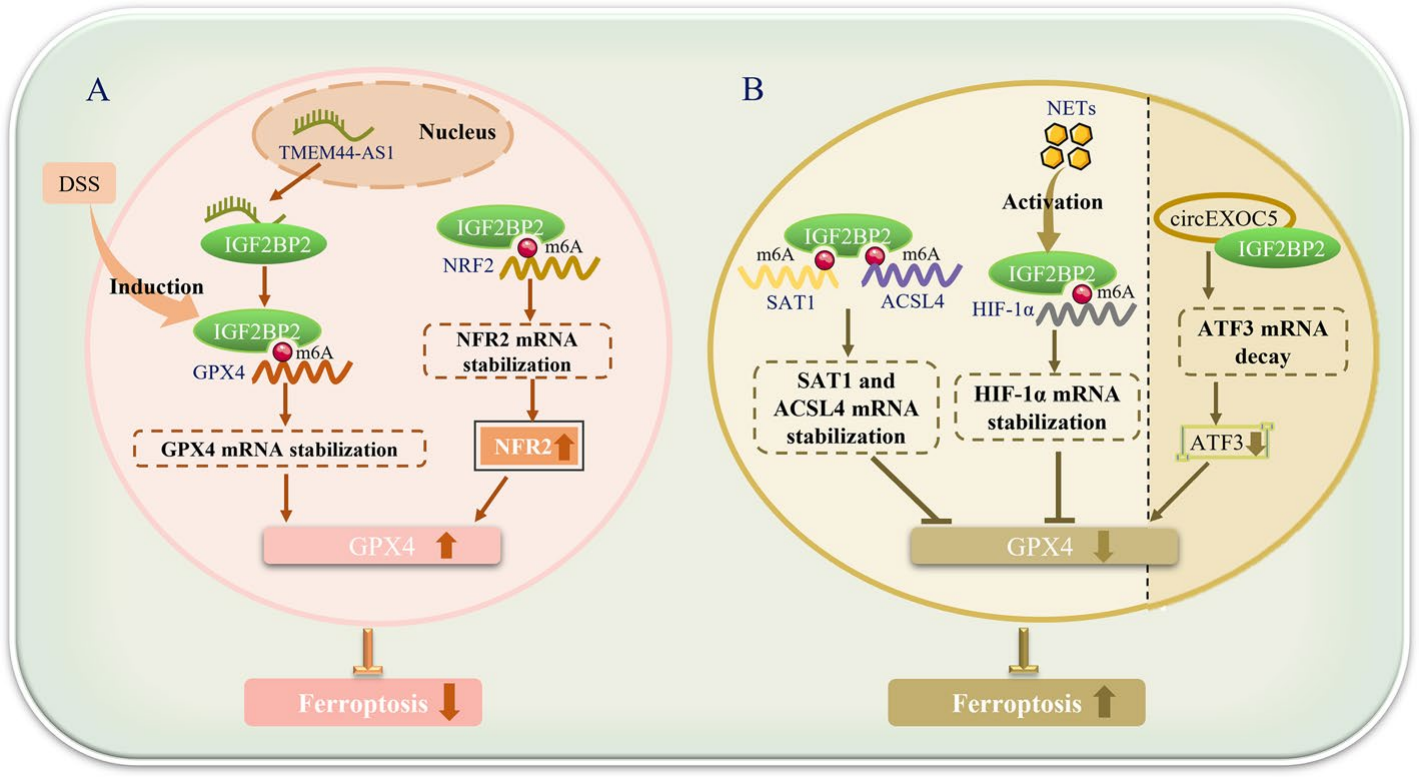
IGF2BP2 affects cell ferroptosis. **A** IGF2BP2 can directly enhance the stability of GPX4 mRNA or indirectly upregulate GPX4 expression by stabilizing NRF2 mRNA through m^6^A modification in human normal colon epithelial cells, ESCC, and HPSCC cells stimulated by sodium dextran sulfate (DSS), thereby inhibiting cell ferroptosis. **B** IGF2BP2 can also downregulate GPX4 expression and promote ferroptosis in alveolar epithelial cells, mouse pancreatic cells, and pulmonary microvascular endothelial cells by enhancing the stability of HIF-1 α, SAT1, and ACSL4 mRNA, or promoting the degradation of ATF3 mRNA through non-m^6^A-modification-dependent mechanisms. *DSS* dextran sulfate sodium salt, *GPX4* glutathione peroxidase 4, *TMEM44-AS1* transmembrane protein 44 antisense RNA 1, *NRF2* nuclear factor erythroid 2-related factor 2, *SAT1* spermidine/spermine N1-acetyltransferase 1, *ACSL4* acyl-CoA synthetase long chain family member 4, *NETs* neutrophil extracellular traps, *HIF-1α* hypoxia-inducible factor 1 subunit alpha, *circEXOC5* circular RNA exocyst complex component 5, *ATF3* activating transcription factor 3. Microsoft PowerPoint was used to create this graphic

GPX4, an antioxidant enzyme, is a downstream mediator of the amino acid antiporter system xc^−^ and can participate in the reduction of phospholipid hydroperoxides in the membrane [30]. Concurrently, GPX4 inhibition and the accumulation of reactive oxygen species (ROS) are closely related to the key feature of iron-dependent lipid peroxidation (LPO) in the process of cell ferroptosis [31]. Currently, a multitude of studies have confirmed that GPX4 plays a significant role in the regulation of cell ferroptosis by IGF2BP2; indeed, IGF2BP2 can directly or indirectly upregulate the expression of GPX4 through m^6^A modification [24–26]. For instance, Liu and colleagues found that IGF2BP2 enhances GPX4 expression through m^6^A modification, thereby inhibiting ferroptosis and attenuating the progression of ulcerative colitis (UC). IGF2BP2 and GPX4 are downregulated in UC treated with dextran sulfate sodium (DSS). Overexpression of IGF2BP2 can enhance the stability of GPX4 mRNA in human normal colon epithelial NCM460 cells stimulated by DSS, thereby inhibiting ROS, malondialdehyde (MDA), and iron levels, ultimately improving UC symptoms, disease activity index scores, inflammatory responses, and ferroptosis [24]. Yang et al. also revealed that lncRNA TMEM44 antisense RNA 1 (TMEM44-AS1) is extremely expressed in ESCC tissues and cells. Mechanistically, TMEM44-AS1 is positively correlated with GPX4 expression. TMEM44-AS1 can bind to the RNA-binding protein IGF2BP2, enhancing the stability of GPX4 mRNA, thereby inhibiting ferroptosis in ESCC cells and accelerating their proliferation, invasion, and metastasis [25]. In addition, Ye et al. found that ALKBH5-mediated m^6^A demethylation inhibits the transcription of nuclear factor erythroid 2-related factor 2 (NRF2). In HPSCC cells, ALKBH5 is poorly expressed, and IGF2BP2 can enhance the stability of NRF2 mRNA through m^6^A modification. Meanwhile, there is a positive correlation between GPX4 levels and NRF2 expression levels. After the expression of NRF2 increases, GPX4 is upregulated, ultimately inhibiting ferroptosis in HPSCC cells and promoting the development of HPSCC [26] (Fig. 1).

In summary, in NCM460 cells stimulated by DSS and tumor cells such as ESCC cells and hypopharyngeal squamous cell carcinoma (HPSCC) cells, IGF2BP2 can directly enhance the stability of GPX4 mRNA through m^6^A modification or indirectly upregulate GPX4 expression by stabilizing NRF2 mRNA, thereby inhibiting cell ferroptosis, improving symptoms of ulcerative colitis (UC), or accelerating the progression of ESCC and hypopharyngeal squamous cell carcinoma. In the gene expression network, long noncoding RNA (lncRNA) serves as an important regulatory factor that can regulate mRNA stability, translation, and posttranslational modifications in the cytoplasm, as well as control nuclear structure and transcription in the nucleus [32]. In the aforementioned study by Yang et al., they found that TMEM44-AS1 can interact with IGF2BP2 by acting as a molecular scaffold between IGF2BP2 and GPX4, ultimately affecting ferroptosis [25]. However, the precise molecular mechanisms by which TMEM44-AS1 functions as a molecular scaffold during this process require further exploration.

### IGF2BP2 enhances mRNA stability of downstream target genes and promotes cell ferroptosis through an m^6^A-modification-dependent mechanism

At the same time, a multitude of studies have also found that, in addition to upregulating the expression of GPX4 and inhibiting cell ferroptosis, IGF2BP2 can also promote ferroptosis in alveolar epithelial cells and mouse pancreatic cells AR42J by enhancing the stability of HIF-1α, spermidine/spermine N1-acetyltransferase 1 (SAT1), and acyl-CoA synthetase long-chain family member 4 (ACSL4) mRNA, than downregulating GPX4 expression, and ultimately exacerbating the inflammatory response in acute lung injury or acute pancreatitis [27–29]. As is well known, during disease progression, polymorphonuclear neutrophils (PMNs) are the first line of immune defense against pathogens [33]. In addition to phagocytosis and degranulation, neutrophils can also release neutrophil extracellular traps (NETs)—DNA material webs decorated with bactericidal proteins—effectively trapping microorganisms in circulation [34]. Zhang et al.’s study showed that, in alveolar epithelial cells of sepsis-induced acute lung injury (SI-ALI) patients, NETs can activate the m^6^A-IGF2BP2-dependent mechanism mediated by methyltransferase-like 3 (METTL3), inducing upregulation of HIF-1α. Subsequently, the level of GPX4 was downregulated, glycolysis was enhanced, and oxidative phosphorylation was reduced, thereby promoting ferroptosis and metabolic reprogramming of alveolar epithelial cells, playing a key role in the progression of SI-ALI [28]. In addition, Chen and colleagues’ research also revealed that, in AR42J cells of severe acute pancreatitis (SAP) mouse pancreas, IGF2BP2 can recognize m^6^A-modified SAT1 and ACSL4 mRNA and enhance their stability. Overexpression of SAT1 or ACSL4 further reduces the levels of GSH, SLC7A11, and GPX4, and upregulates intracellular Fe^2+^ levels, ultimately exacerbating SAP inflammation by promoting cell ferroptosis [29] (Fig. 1).

### IGF2BP2 accelerates ATF3 mRNA degradation and promotes cell ferroptosis through a non-m^6^A-modification-dependent mechanism

Numerous studies have shown that IGF2BP2, as an m^6^A reader, primarily functions by enhancing the mRNA stability of downstream target genes. However, Wang et al. found that IGF2BP2 can also affect the degradation of target gene mRNA through mechanisms that are independent of m^6^A modification. In primary microvascular endothelial cells (PMVECs), the demethylase ALKBH5 can stabilize the expression of circular RNA exocyst complex component 5 (circEXOC5) through demethylation, which in turn recruits IGF2BP2 to promote the degradation and shorten the half-life of ATF3 mRNA, ultimately promoting ferroptosis in PMVECs and exacerbating sepsis-induced acute lung injury (ALI) [27] (Fig. 1).

Today, with m^6^A modification becoming a hot topic, IGF2BP2 has received considerable attention as an m^6^A reader whose primary function is to enhance the stability of downstream target gene mRNA. However, in the aforementioned studies, IGF2BP2 also played a crucial role in the degradation of ATF3 mRNA. In addition, recent studies have found that, in triple-negative breast cancer (TNBC) cells, IGF2BP2 can upregulate the protein level of cyclin-dependent kinase 6 (CDK6) by regulating the shift in translation rate during the initiation of CDK6 translation, rather than the stability of mRNA [35]. This suggests that, in addition to its impact on downstream target gene mRNA stability, IGF2BP2 also has many potential roles that need to be explored, such as its effects on mRNA translation rates or protein levels.

## IGF2BP2 promotes EMT in tumor cells

EMT is a cellular process by which epithelial cells lose some of their epithelial characteristics and acquire a mesenchymal phenotype to facilitate cell movement [36]. An increasing body of evidence suggests that the abnormal activation of the EMT developmental program contributes to the occurrence, invasion, metastasis, and acquisition of treatment resistance in tumors [37, 38]. In tumor cells, various signals from the tumor microenvironment can trigger the activation of EMT, such as Wnt, Notch, growth factor receptor signaling, and inflammatory cytokines [39, 40]. The induction of EMT is coordinated by numerous core EMT transcription factors (TFs), including snail family transcriptional repressors 1 and 2 (SNAI1/2), twist family bHLH transcription factors 1 and 2 (TWIST1/2), and zinc finger E-box binding homeobox 1 and 2 (ZEB1/2) [37]. Wang et al. summarized that IGF2BP2 can disrupt TGF-β-induced EMT in lung cancer cells, and miR-138 inhibits EMT in glioma cells by targeting IGF2BP2 at the 3′-UTR level [30, 31]. However, in recent years, research on the impact of IGF2BP2 on the EMT process has been continuously reported. Here, we update the relevant research showing that IGF2BP2 promotes EMT in tumor cells and accelerates disease progression by affecting the mRNA stability of genes related to the EMT process. In addition, we provide a detailed review of the molecular mechanisms by which IGF2BP2 promotes EMT in tumor cells under the influence of upstream regulatory factors [32–38].

### IGF2BP2 promotes the EMT process of tumor cells by affecting the mRNA stability of its downstream target genes

**Fig. 2:**
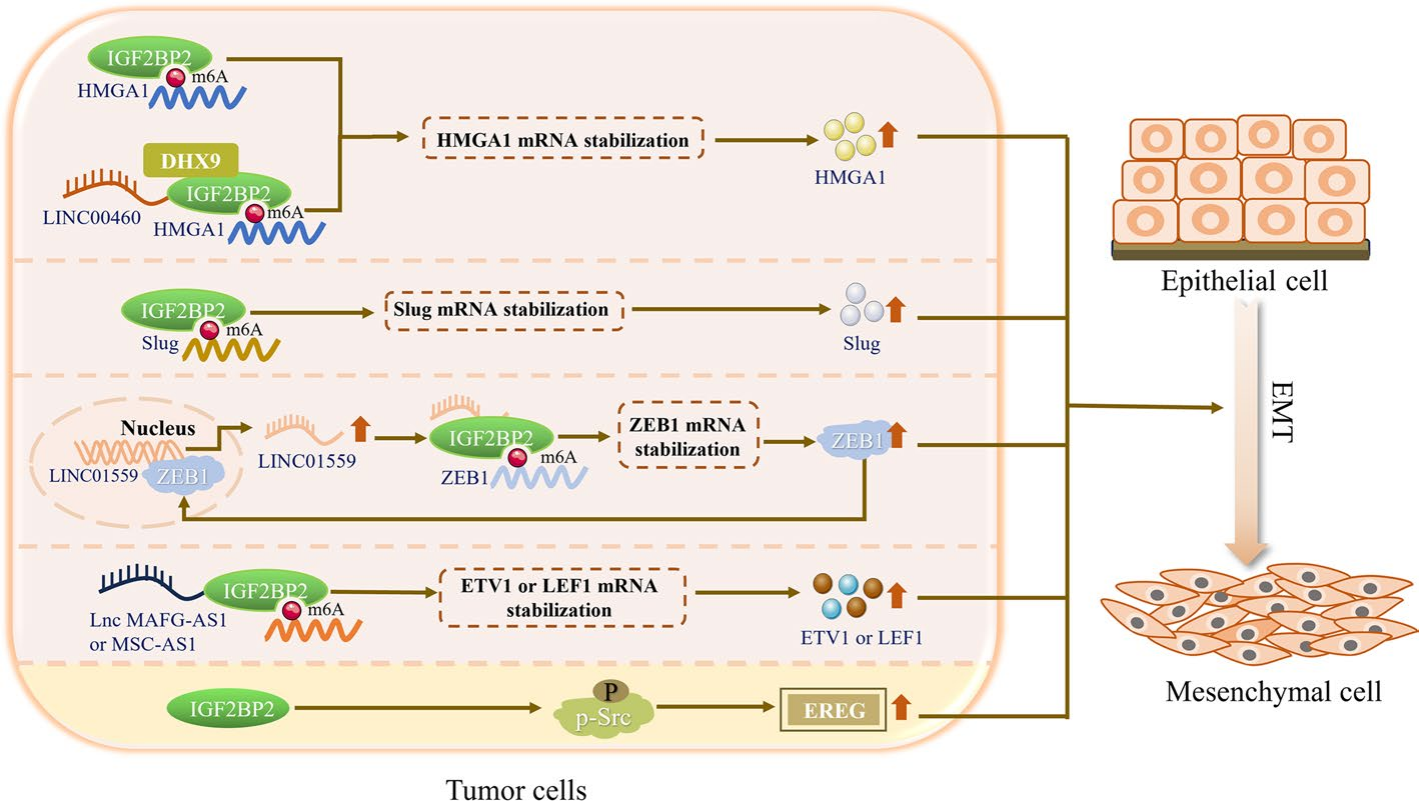
IGF2BP2 promotes EMT process in tumor cells. In tumor cells such as gastric cancer and colorectal cancer, IGF2BP2 can promote the stability of Slug, HMGA1, and ZEB1 mRNA by directly recognizing and binding to their m^6^A site or being recruited by upstream regulatory factors, ultimately promoting the EMT process of tumor cells. In addition, IGF2BP2 can also upregulate EREG genes and trigger EMT in head and neck cancer by promoting phosphorylation levels of Src and other genes instead of m^6^A modification dependence. *HMGA1* high mobility group A1, *DHX9* DExH-box helicase 9, *LINC00460* long intergenic non-protein-coding RNA 460, *LINC01559* long intergenic non-protein-coding RNA 1559, *ZEB1* zinc finger E-box binding homeobox 1, *Lnc MAFG-AS1* MAF BZIP transcription factor G antisense RNA 1, *MSC-AS1* musculin antisense RNA 1, *ETV1* ETS translocation variant 1, *LEF1* lymphoid enhancer binding factor 1, *Src* nonreceptor tyrosine kinase gene, *EREG* epiregulin. Microsoft PowerPoint was used to create this graphic

Slug belongs to the snail family of zinc finger transcription inhibitors, and its most studied functions include regulating epithelial cell plasticity and EMT [41]. It is indispensable for tissue maintenance and tumor progression [42–45]. High mobility group A1 (HMGA1) protein is a small nuclear protein that can serve as a structural transcription factor [46]. It has been shown to activate multiple genes involved in tumorigenesis, tumor proliferation, migration, invasion, and EMT [47]. There is currently evidence to suggest that IGF2BP2 can enhance the stability of Slug and HMGA1 mRNA through m^6^A-modification-dependent mechanisms and play a role in the EMT process of tumor cells such as head and neck squamous cell carcinoma (HNSCC) and gastric cancer (GC) [48–50]. For example, Yu et al. confirmed that Slug is a key EMT-related transcription factor in HNSCC tissue and a direct target of IGF2BP2. IGF2BP2 can bind to the m^6^A site in the Slug coding sequence (CDS) region, promoting the stability of its mRNA and ultimately facilitating lymph node metastasis, lymphangiogenesis, as well as HNSCC cell migration and invasion through the EMT process in vivo [48]. Ouyang et al.’s experiment also found that the expression of IGF2BP2 was significantly upregulated in GC tissues. IGF2BP2 enhances its stability by directly interacting with HMGA1 mRNA, thereby increasing its expression, promoting GC cell migration and invasion, as well as EMT [49]. In addition, Hou et al.’s study also revealed that IGF2BP2 can interact with DExH-box helicase 9 (DHX9) and long intergenic non-protein-coding RNA 460 (LINC00460) in colorectal cancer (CRC) cells, thereby binding to the 3′-UTR of HMGA1 mRNA, enhancing its mRNA stability, and ultimately inducing the EMT of CRC cells, promoting their proliferation, migration, and invasion [50] (Fig. 2).

Overall, in HNSCC, GC, and CRC cells, IGF2BP2 can directly recognize and bind to the m^6^A sites of Slug and HMGA1 mRNAs, or interact with DHX9 and LINC00460 to promote the stability of Slug and HMGA1 mRNA, ultimately facilitating the EMT of tumor cells and accelerating their invasion and migration [48–50].

### The upstream regulatory factors of IGF2BP2 affect its function, thereby accelerating the EMT process of tumor cells

In addition to directly or indirectly acting on Slug and HMGA1, IGF2BP2 may also be recruited by various noncoding RNAs, thereby stabilizing downstream target gene mRNAs such as ZEB1 and Cell division cycle 27 (CDC27), and playing a role in the EMT process of tumor cells [51–54]. For example, Shen et al. confirmed that, in GC cells, ZEB1 acts as a transcription factor that binds to the long intergenic non-protein-coding RNA 1559 (LINC01559) promoter, activating the expression of LINC01559. Meanwhile, LINC01559 can recruit IGF2BP2 to stabilize ZEB1 mRNA and upregulate ZEB1 expression in GC cells. The formation of the ZEB1/LINC01559/IGF2BP2 positive feedback loop ultimately accelerates GC cell proliferation, migration, and EMT processes [51]. Zhao et al. also found that, in hepatocellular carcinoma (HCC) cells, transcription factor 7-like 2 (TCF7L2) promotes the transcription of circ_0000775 and induces its high expression. Subsequently, circ_0000775 maintains the stability of CDC27 mRNA by recruiting IGF2BP2, thereby positively regulating the expression of CDC27 and ultimately promoting HCC cell migration, invasion, and EMT [52]. At the same time, the research results of Weng et al. and Ma et al. also showed that, in pancreatic cancer (PC) and melanoma cells, lncRNA MAF BZIP transcription factor G antisense RNA 1 (MAFG-AS1) and musculin antisense RNA 1 (MSC-AS1) can also stabilize ETS translocation variant 1 (ETV1) and lymphoid enhancer binding factor 1 (LEF1) mRNA, respectively, by recruiting IGF2BP2, and ultimately promote tumor cell migration, invasion, proliferation, and EMT [53, 54]. In summary, in GC, HCC, PC, and melanoma cells, IGF2BP2 can be recruited by upstream regulatory factors LINC01559, circ_0000775, lncRNA MAFG-AS1, and MSC-AS1 to stabilize ZEB1, CDC27, ETV1, and LEF1 mRNA, respectively. Ultimately, this promotes EMT of tumor cells and accelerates their migration and invasion (Fig. 2).

At the same time, researchers have revealed that, in HNSCC, IGF2BP2 can also activate the nonreceptor tyrosine kinase Src signaling pathway by promoting the phosphorylation levels of focal adhesion kinase (FAK), Raf, Src, and mitogen-activated protein kinase kinase (MEK), thereby mediating the upregulation of the epiregulin (EREG) gene. Ultimately, this triggers EMT, promoting the migration and invasion response of oral squamous cell carcinoma (OSCC) cells [55] (Fig. 2). This study expands our understanding of the role of IGF2BP2 in phosphorylation, which is beneficial for a comprehensive understanding of the role and molecular mechanisms of IGF2BP2. However, this study also has limitations. For instance, in the process of IGF2BP2 regulating EMT in HNSCC cells, how does IGF2BP2 promote the phosphorylation level of downstream target genes, and what is the specific molecular mechanism? Besides promoting the phosphorylation levels of genes such as FAK and Raf, is the m^6^A modification-dependent mechanism involved in regulating the expression of EREG? These issues warrant further exploration.

## IGF2BP2 promotes the stemness characteristics of tumor cells and hematopoietic stem cells

Stem cells are a unique type of cell that can not only divide to produce more cells with stemness but also differentiate into other types of cells [56, 57]. Compared with terminally differentiated cells, stem cells retain the ability to reenter the cell cycle and proliferate [58]. Moreover, the sustained existence of stem cell states may also lead to the production of cancer stem cells (CSCs). CSCs exhibit preferential invasiveness and are closely related to the occurrence, spread, and treatment resistance of cancer [59–61]. In recent years, the relationship between IGF2BP2 and cell stemness has also attracted widespread attention. Wang et al. mentioned that IGF2BP2 can promote stem cell-like characteristics of pancreatic cancer (PC) cells by binding to and stabilizing m^6^A-modified DANCR RNA [53]. On this basis, we have updated and summarized the specific molecular mechanisms by which IGF2BP2 stabilizes mRNA of downstream target genes such as SOX2 and CCAR1, enhances the stemness characteristics of colorectal cancer (CRC) cells and hematopoietic stem cells, and ultimately affects disease progression [53–59].

### IGF2BP2 regulates the mRNA stability of its downstream target genes, thereby promoting the stemness of tumor cells

In many types of tumors, fundamental tumor characteristics such as proliferation and invasion are related to cell stemness [62–65]. Currently, there is evidence suggesting that IGF2BP2 plays a role in the development of various tumor cell stemness through m^6^A-modification-dependent mechanisms, ultimately affecting disease progression [66–71]. For example, Weng et al. also observed that IGF2BP2 plays an important role in the self-renewal of leukemia stem cells/initiating cells (LSCs/LICs). IGF2BP2 is highly expressed in LSCs/LICs, and silencing of IGF2BP2 or depletion of METTL3 and METTL14 can lead to significant reprogramming of cellular metabolites, especially those involved in glutamine (Gln) and glutamate (Glu) metabolism. Mechanistically, IGF2BP2 interacts with the components of the eukaryotic translation initiation factor (eIF) complex. It recruits eIF proteins to its target mRNA and regulates the expression of key targets V-myc avian myelocytomatosis viral oncogene homolog (MYC), glutamic-pyruvic transaminase 2 (GPT2), and solute carrier family 1 member 5 (SLC1A5) in the glutamine metabolism pathway in an m^6^A-modification-dependent manner, ultimately promoting the development of acute myeloid leukemia (AML) and self-renewal of leukemia stem cells/initiating cells [67]. Li et al. also confirmed that the expression of sex-determining region Y-box 2 (SOX2) is positively correlated with METTL3 and IGF2BP2 in CRC tissues. Methylated SOX2 transcripts, especially the CDS region, are specifically recognized by IGF2BP2 to enhance their stability, ultimately promoting self-renewal and cell stemness in CRC [68]. In addition, Huang et al. also discovered m^6^A modification of circABCC4 mediated by METTL3 in prostatic carcinoma (PCa). Subsequently, m^6^A-circABCC4 recruited IGF2BP2 protein to cell division cycle and apoptosis regulator 1 (CCAR1) mRNA, thereby enhancing the stability of CCAR1 mRNA and activating the Wnt/β-catenin pathway, ultimately promoting the stemness and metastasis of PCa cells [69]. Overexpression of IGF2BP2 in HCC and ESCC cells can also respectively stabilize cell division cycle 45 (CDC45) and octamer-binding transcription factor 4 (OCT4) mRNA through m^6^A modification, ultimately promoting tumor cell stemness and exerting tumorigenic effects [70, 71]. In summary, in tumor cells such as PC, leukemia, CRC, and PCa, IGF2BP2 enhances cellular stemness by stabilizing specific mRNAs, including SOX2, and CCAR1, among others. This stabilization promotes tumor cell proliferation and migration, ultimately accelerating cancer progression.

It is well established that IGF2BP2 functions in recognizing and binding to the m^6^A site, without engaging in editing. Therefore, during the process where m^6^A levels rise and mRNA fate is influenced, there often exists a “cooperative” relationship between the m^6^A reader and writer. The collaboration between IGF2BP2 and m^6^A writers (e.g., METTL3) has been described in the studies by Weng, Li, and Huang et al., as mentioned above [67–69]. The nuclear translocation of METTL3 is crucial for m^6^A modification, and its subcellular distribution serves as a fundamental factor in determining its physiological functions [72]. Li et al. discovered that a positive feedback loop dependent on IL-6 m^6^A modification promotes the function of nuclear METTL3. This loop also influences the subcellular localization of METTL3, ultimately contributing to breast cancer (BC) metastasis [73]. The mRNA transcript of IL-6 is modified by m^6^A mediated by METTL3, which enhances the stability of IL-6 mRNA. Subsequently, IL-6 induced deacetylation of K177-METTL3 promotes nuclear transfer of METTL3 through the AMPK/SIRT1 axis, which plays a crucial role in its m^6^A modification. In conclusion, this positive feedback loop, which depends on METTL3-mediated IL-6 m^6^A modification, enhances the migration and invasion potential of BC cells. Typically, IGF2BP2 enhances the stability of specific mRNAs by recognizing their m^6^A modifications. An intriguing question then arises: does IGF2BP2 play a role in the process by which IL-6 promotes the nuclear translocation of METTL3? The answer likely hinges on whether IGF2BP2 assists in enhancing the stability of IL-6 mRNA that is mediated by METTL3-m^6^A. Presently, this remains an open question.

As another set of crucial proteins involved in m^6^A biological processes, the demethylases—referred to as “erasers”—are another important group of proteins involved in m^6^A biological processes, with ALKBH5 and FTO being the most well-known examples. In recent years, the relationship between IGF2BP2 and m^6^A erasers has been extensively studied. A multitude of studies have revealed that m^6^A erasers exhibit low expression levels in tumor cells such as HPSCC, CRC, and papillary thyroid cancer (PTC). This downregulation appears to enhance the ability of m^6^A readers such as IGF2BP2 to recognize m^6^A modifications [74, 75]. For instance, downregulation of FTO and ALKBH5 collaboratively activate FOXO signaling through m^6^A modification of HK2 mRNA mediated by IGF2BP2. As a result, glycolysis in CRC is enhanced, thereby accelerating the malignant biological behavior of tumor cells [74]. Additionally, FTO inhibits glycolysis and growth in PTC cells by blocking IGF2BP2 recognition of m^6^A modifications and reducing the stability of APOE mRNA [75]. Nevertheless, in the aforementioned studies exploring the effect of IGF2BP2 on the stemness of tumor cells, it remains unclear whether FTO and ALKBH5 are consistently downregulated to support the function of IGF2BP2. Furthermore, it is an open question whether FTO and ALKBH5 can regulate IGF2BP2 function through other potential mechanisms beyond m^6^A modification. These questions highlight the need for further in-depth research to elucidate the complex interactions between m^6^A erasers and IGF2BP2 in cancer progression. These issues still warrant further in-depth research.

### IGF2BP2 regulates the mRNA stability of downstream target genes, promotes self-renewal of hematopoietic stem cells, and maintains their function

Hematopoietic stem cells (HSCs) possess extensive self-renewal capabilities and multilineage differentiation potential, which are essential for the continuous production of blood cells [76]. In adults, HSCs reside within a specialized bone marrow microenvironment that comprises various hematopoietic and non-hematopoietic cell types, including mesenchymal stem cells and endothelial cells [77, 78]. Research by Yin et al. has demonstrated that elevated expression of m^6^A-IGF2BP2 regulates the transcriptional state and sustains the maintenance of HSCs, with IGF2BP2 being crucial for preserving HSC function. Mechanistically, a deficiency in IGF2BP2 significantly reduces the stability of Bmi1 mRNA within HSCs. Bmi1 is vital for modulating mitochondrial function and sustaining HSCs [79, 80]. The accelerated degradation of Bmi1 mRNA results in increased expression of mitochondrial-related genes, indicating heightened mitochondrial activity in HSCs. This, in turn, leads to the loss of HSC quiescence and a subsequent impairment of their function [81]. From a certain perspective, m^6^A can be regarded as a quality control mechanism for maintaining HSC integrity. This study provides the first clear evidence that the m^6^A reader IGF2BP2 is a pivotal factor in safeguarding hematopoietic stem cell stemness and elucidates its role and molecular mechanisms in the maintenance of HSCs and hematopoiesis.

## IGF2BP2 promotes angiogenesis

Angiogenesis, which is the process of forming new blood vessels, is a complex and dynamic process. It is regulated by a variety of pro-angiogenic and anti-angiogenic molecules and plays a pivotal role in tumor growth, invasion, and metastasis [82]. With the advancement of molecular and cellular biology, various biomolecules involved in tumor angiogenesis, including growth factors, chemokines, and adhesion molecules, have been progressively identified [69–71]. For nearly 3 years, a large number of studies have revealed that IGF2BP2 also exerts a significant influence on angiogenesis in diseases such as tumors and retinopathy [72–79]. Therefore, it is necessary to timely summarize the effects and molecular mechanisms of IGF2BP2 on angiogenesis.

### IGF2BP2 enhances the mRNA stability of downstream target genes through an m^6^A-modification-dependent mechanism, thereby promoting angiogenesis

**Fig. 3:**
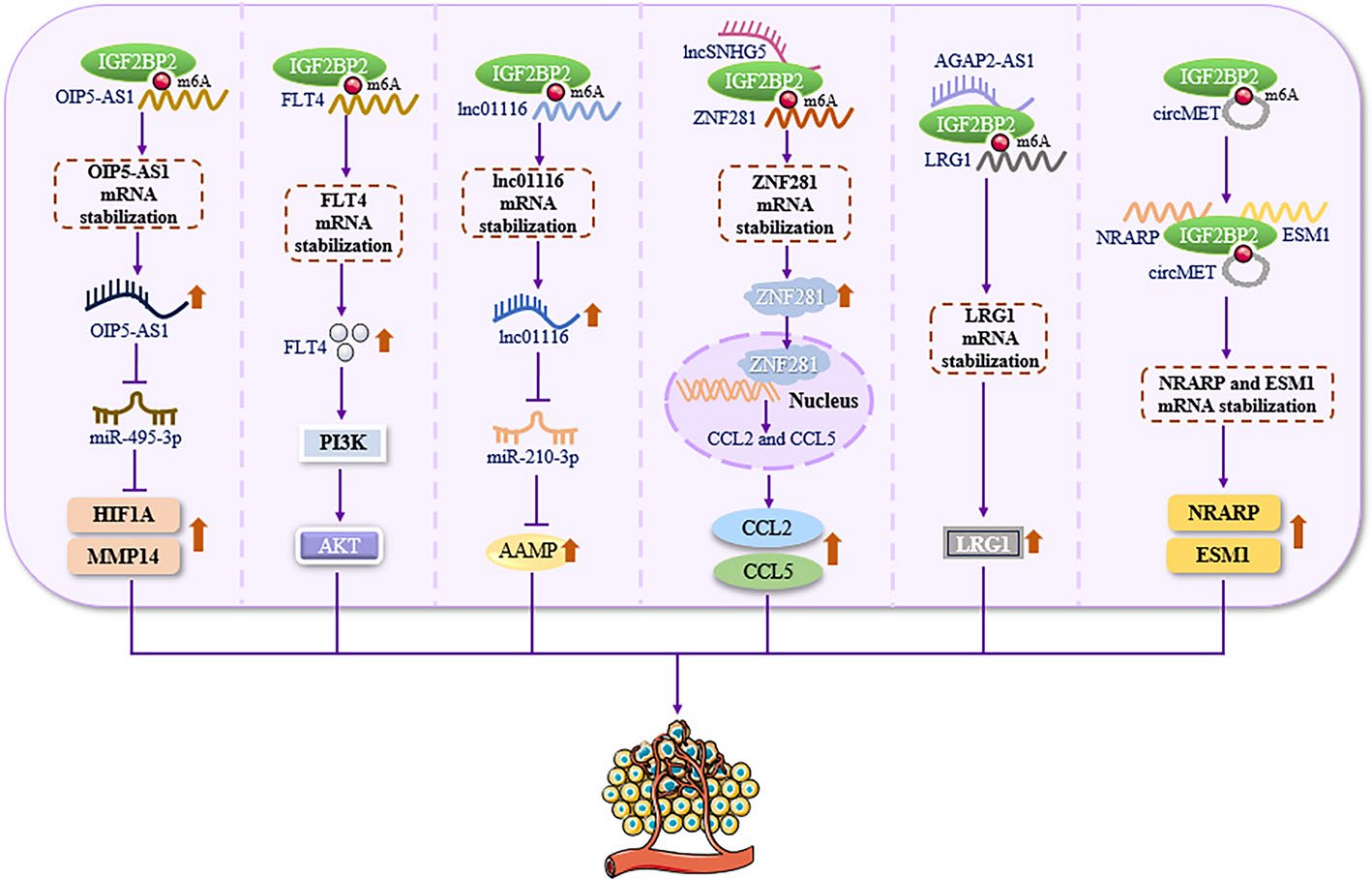
IGF2BP2 promotes angiogenesis. IGF2BP2 can enhance the stability of OIP5-AS1, FLT4, and lnc0116 mRNA in an m^6^A-modification-dependent manner or bind to lncSNHG5, AGAP2-AS1, and circMET, respectively, to enhance the stability of ZNF281, LRG1, and NRARP/ESM1 mRNA in nourishing cells and tumor cells such as lung adenocarcinoma and glioma, ultimately promoting angiogenesis. *OIP5-AS1* opa interacting protein 5 antisense RNA 1, *miR-495-3p* microRNA-495-3p, *HIF1A* hypoxia-inducible factor 1 subunit alpha, *MMP14* matrix metallopeptidase 14, *FLT4* Fms-related receptor tyrosine kinase 4, *PI3K* phosphatidylinositol-3-kinase, *AKT* protein kinase B, *lnc01116* long intergenic non-protein-coding RNA 1116, *miR-210-3p* microRNA-210-3p, *AAMP* angio-associated migratory cell protein, *lncSNHG5* long noncoding RNA small nucleolar RNA host gene 5, *ZNF281* zinc finger protein 281, *CCL2* C–C motif chemokine ligand 2, *CCL5* C–C motif chemokine ligand 5, *AGAP2-AS1* AGAP2 antisense RNA 1, *LRG1* leucine-rich α-2 glycoprotein 1, *circMET* circular RNA MET, *NRARP* Notch regulated ankyrin repeat protein, *ESM1* endothelial cell specific molecule 1. Microsoft PowerPoint was used to create this graphic

Research has demonstrated that IGF2BP2 plays a role in regulating tumor development by modulating the proliferation, invasion, and migration of various human cancer cells, including colorectal and lung cancer cell lines. As the significance of angiogenesis in tumor growth becomes increasingly recognized, IGF2BP2 has garnered considerable interest from researchers owing to its potential to promote tumor angiogenesis and accelerate cancer progression [83, 84]. For instance, Li et al. confirmed that IGF2BP2 can enhance the stability of the opa interacting protein 5 antisense RNA 1 (OIP5-AS1) mRNA in glioma tissues and cells. This enhancement increases the binding of OIP5-AS1 to MicroRNA-495-3p (miR-495-3p), ultimately promoting the formation of vasculogenic mimicry (VM) in glioma [83]. Concurrently, Fang et al. discovered that IGF2BP2 is specifically expressed in subsets of lung adenocarcinoma (LUAD) cells (referred to as LUAD_SGF2BP2) and endothelial cells (referred to as En_SGF2BP2). IGF2BP2 is preferentially secreted by exosomes from the LUAD_SGF2BP2 subset and taken up by the En_SGF2BP2 subset within the tumor microenvironment. Subsequently, IGF2BP2 enhances the stability of Fms-related receptor tyrosine kinase 4 (FLT4) mRNA through m^6^A modification, thereby activating the phosphatidylinositol-3-kinase (PI3K)/protein kinase B (Akt) signaling pathway. This activation ultimately promotes LUAD angiogenesis and metastasis [84] (Fig. 3).

In addition to its role in tumor angiogenesis, IGF2BP2 has also been identified as a promoter of angiogenesis in trophoblast and wound healing contexts [85, 86]. Zhang et al. have shown that IGF2BP2 can modulate the stability of the long intergenic non-protein-coding RNA 1116 (lnc01166) mRNA through m^6^A methylation in trophoblast cells. This regulation leads to an upregulation of the angio-associated migratory cell protein (AAMP) expression by sequestering microRNA-210-3p (miR-210-3p), which in turn promotes trophoblast angiogenesis and may inhibit the development of preeclampsia [85]. Furthermore, research by Zhi and colleagues suggests that IGF2BP2 could emerge as a novel therapeutic target for conditions such as diabetes, aging, and vascular diseases. In keratinocytes (HaCaT), IGF2BP2 can bind to the 3′-UTR of heparinase (HPSE) mRNA, thereby stabilizing it and ultimately promoting the proliferation and migration of HaCaT cells. This process accelerates wound healing and angiogenesis [86]. In summary, IGF2BP2 can enhance the stability of various mRNAs, including lnc01116, HPSE, FLT4, and OIP5-AS1, in different cell types such as keratinocytes, trophoblasts, lung adenocarcinoma, and glioma. This enhancement promotes angiogenesis, potentially inhibits preeclampsia, accelerates wound healing, and contributes to the development of LUAD, glioma, and other tumors, respectively. However, the in vivo data from Zhi et al. are lacking, and further in vivo studies are needed to explore the expression pattern of IGF2BP2 and its role in promoting angiogenesis (Fig. 3).

### IGF2BP2 upstream regulators affect its function and promote angiogenesis

As an RBP, IGF2BP2 has been shown to interact with long noncoding RNAs (lncRNAs) such as Notch regulated ankyrin repeat protein (NRARP) and endothelial cell specific molecule 1 (ESM1). This interaction modulates the posttranscriptional fate of downstream genes, enhances mRNA stability, and promotes angiogenesis in conditions such as bladder cancer (BCa), breast cancer (BC), and retinopathy [87–89]. For instance, Zhao et al. discovered that IGF2BP2 and AGAP2 antisense RNA 1 (AGAP2-AS1) are highly upregulated in BCa. AGAP2-AS1, predominantly found in the cytoplasm, can bind directly to IGF2BP2, thereby stabilizing leucine-rich α-2 glycoprotein 1 (LRG1) mRNA and promoting tumor proliferation, migration, invasion, and angiogenesis in vitro, as well as tumor growth and metastasis in vivo [87]. Zeng and colleagues confirmed that IGF2BP2 can stabilize zinc finger protein 281 (ZNF281) mRNA by binding to the long noncoding RNA small nucleolar RNA host gene 5 (lncSNHG5). This interaction leads to the upregulation of C–C motif chemokine ligand 2 (CCL2) and C–C motif chemokine ligand 5 (CCL5), activating the p38 mitogen-activated protein kinase (P38 MAPK) signaling in endothelial cells and promoting angiogenesis and vascular leakage, which can accelerate tumor metastasis and lead to poor prognosis in BC patients [88]. Additionally, Yao et al. found that, in diabetic retinopathy, circular RNA MET (circMET) interacts with IGF2BP2 in endothelial cells. Overexpression of IGF2BP2 significantly increases the expression of m^6^A-modified circMET. Subsequently, circMET forms a complex with IGF2BP2 and either NRARP or ESM1, enhancing their mRNA stability and promoting retinal angiogenesis and accelerating retinal lesions [89] (Fig. 3). In summary, by binding to AGAP2-AS1, lncSNHG5, and circMET, IGF2BP2 enhances the stability of ZNF281, NRARP, and ESM1 mRNA in a manner dependent on m^6^A modification. This enhancement promotes angiogenesis in BCa, BC, and retinopathy, respectively, and can accelerate disease progression.

## IGF2BP2 affects inflammatory response

Tumor-associated inflammation (TAI) is a hallmark of most cancers, exerting both promoting and inhibiting effects on tumorigenesis. Inflammatory cytokines and cells are implicated in the development and progression of tumors across various body sites. Monocytes, the precursors of macrophages, are derived from hematopoietic stem cells, enter the bloodstream, and migrate to peripheral tissues, where they differentiate into macrophages and dendritic cells [90]. As key immune cells in the tumor microenvironment, macrophages play a pivotal role in TAI development [91, 92]. Influenced by the local microenvironment and pathophysiological factors, macrophages can polarize into two distinct phenotypes: pro-inflammatory macrophages (M1) and anti-inflammatory macrophages (M2) [93]. Inflammatory cytokines are vital in normal cellular inflammation, host immune responses, and cell growth regulation, and they are central mediators in many autoimmune and chronic inflammatory diseases [94]. A multitude of inflammatory cytokines are also linked to cancers such as BC, GC, and LUAD [95]. Over the past 3 years, IGF2BP2 has been widely reported to influence macrophage polarization and regulate cytokine secretion, thereby modulating the inflammatory response and promoting disease progression. Consequently, we have summarized the relevant studies showing that IGF2BP2 affects the inflammatory response by enhancing the stability of mRNA from downstream target genes [86–94].

### IGF2BP2 affects inflammatory response by influencing macrophage polarization or regulating cytokine secretion

#### IGF2BP2 inhibits inflammatory response by promoting polarization of M2 macrophages

M2 macrophages predominantly secrete anti-inflammatory cytokines, such as interleukin-10 (IL-10), which aid in clearing receptors and various remodeling factors. This process helps to reduce inflammation, dampen the immune response, and facilitate tissue repair and remodeling [96, 97]. In the context of tumors, tumor-associated macrophages (TAMs) are often induced by environmental cues to adopt the M2 macrophage phenotype. This adoption contributes to the establishment of an immunosuppressive tumor niche, which can promote tumor progression and resistance to therapy [98, 99].

**Fig. 4:**
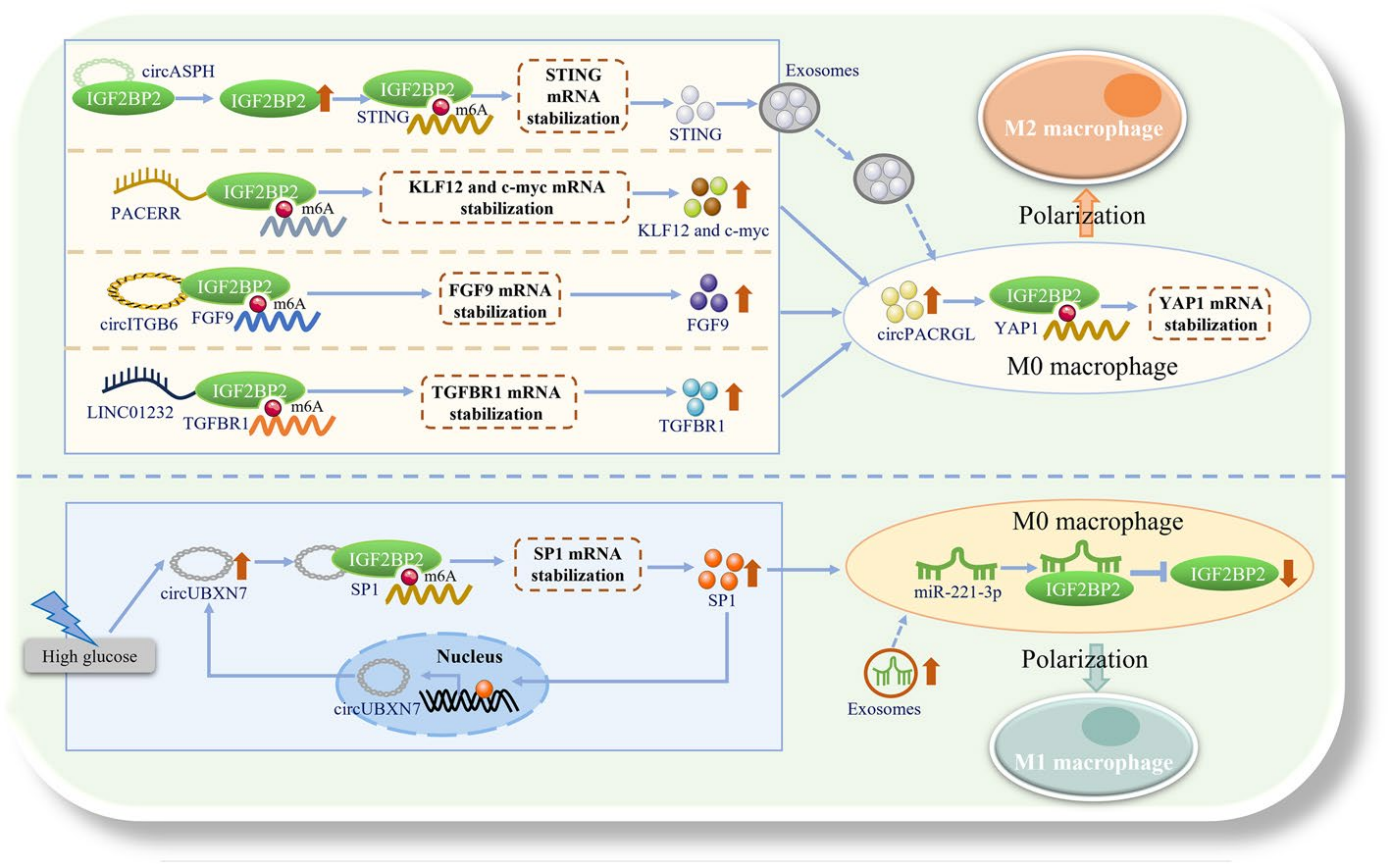
IGF2BP2 affects macrophage polarization. Under the action of circASPH, LncRNA PACERR, circITGB6, and LINC01232 in tumor cells such as CRC, PDAC, OC, and non-small cell lung cancer (NSCLC), IGF2BP2 enhances the stability of STING, KLF12, c-myc, FGF9, and TGFBR1 mRNA, ultimately promoting polarization of M2 macrophages. Meanwhile, IGF2BP2 in macrophages can enhance the stability of YAP1 mRNA and promote polarization of M2 macrophages under the action of circPACRGL. In addition, in human renal tubular epithelial cells, the binding of IGF2BP2 to circUBXN7 can enhance the stability of SP1 mRNA and promote polarization of M1 macrophages through m^6^A modification. At the same time, IGF2BP2 in macrophages is downregulated by miR-221-3p, ultimately promoting polarization of M1 macrophages. *circASPH* circular RNA aspartate beta-hydroxylase, *STING* stimulator of interferon genes, *PACERR* PTGS2 antisense NFKB1 complex-mediated expression regulator RNA, *KLF12* Krüppel-like factor 12, *c-myc* transcriptional regulator Myc-like, *circITGB6* circular RNA integrin subunit beta 6, *FGF9* fibroblast growth factor 9, *LINC01232* long intergenic non-protein-coding RNA 1232, *TGFBR1* transforming growth factor beta receptor 1, *circPACRGL* circular RNA Parkin coregulated like, *YAP1* Yes1-associated transcriptional regulator, *circUBXN7* circular RNA UBX domain protein 7, *SP1* Sp1 transcription factor, *miR-221-3p* microRNA-221-3p. Microsoft PowerPoint was used to create this graphic

The notion that IGF2BP2-m^6^A in tumor cells promotes the polarization of M2 macrophages, thereby accelerating cancer and inflammatory diseases, has been substantiated by numerous studies [100–103]. For instance, Li and Zhang and their teams discovered that circular RNA integrin subunit beta 6 (circITGB6) and circular RNA aspartate beta-hydroxylase (circASPH) can directly interact with IGF2BP2 in ovarian cancer (OC) and CRC cells, stabilizing IGF2BP2 protein. IGF2BP2 then enhances the stability of fibroblast growth factor 9 (FGF9) and stimulator of interferon genes (STING) mRNA, inducing TAM polarization toward M2 macrophages. This process promotes M2 macrophage-dependent cisplatin (CDDP) resistance in OC and accelerates CRC progression [102, 103]. Furthermore, Liu, Zhu, and colleagues confirmed that IGF2BP2, when bound to lncRNA PTGS2 antisense NFKB1 complex-mediated expression regulator RNA (PACERR) and long intergenic non-protein-coding RNA 1232 (LINC01232), can enhance the stability of Krüppel-like factor 12 (KLF12), transcriptional regulator Myc-like (c-myc), and transforming growth factor beta receptor 1 (TGFBR1) mRNA in the cytoplasm through m^6^A modification. This enhancement promotes M2 macrophage polarization and accelerates the progression of pancreatic ductal adenocarcinoma (PDAC) and NSCLC, respectively [100, 101] (Fig. 4).

Studies have also shown that IGF2BP2 in macrophages can regulate their own gene expression, promote M2 macrophage polarization, and accelerate disease progression [99, 104]. For example, Liu et al. demonstrated that exosomes derived from non-small cell lung cancer (NSCLC) can transfer circular RNA Parkin coregulated-like (circPACRGL) to human monocytic leukemia cells (THP-1). CircPACRGL then binds to IGF2BP2, enhancing the stability of Yes1-associated transcriptional regulator (YAP1) mRNA. This interaction modulates Hippo signaling in THP-1 cells, promotes M2 macrophage polarization, and ultimately accelerates the proliferation, migration, and invasion of NSCLC cells [104] (Fig. 4). Similarly, Wang et al. found that IGF2BP2 expression is higher in bone marrow-derived macrophages and peritoneal macrophages. During cockroach allergen (CRE)-induced allergic inflammation, IGF2BP2 directly binds to tuberous sclerosis complex 1 (TSC1) and peroxisome proliferator-activated receptor γ (PPARγ). This binding enhances the stability of TSC1 and PPARγ, activating the TSC1/mechanistic target of rapamycin complex 1 (mTORC1) pathway and PPARγ-mediated fatty acid uptake. These actions shift M0 macrophages toward M2 activation, exacerbating pulmonary inflammation [99]. Additionally, Lin et al. confirmed that, in macrophages, circADAMTS6 stabilizes CAMK2A mRNA by forming a ternary complex with IGF2BP2 and CAMK2A. This interaction leads to CREB activation, driving M2 macrophage polarization and accelerating emphysema progression [105].

In the current literature, the primary mechanism for regulating IGF2BP2 is through modulating its expression levels, which in turn affects IGF2BP2’s ability to recognize and bind to its target gene mRNAs. Future research may focus on the efficiency of upstream regulatory factors that directly influence IGF2BP2’s recognition and binding to these mRNAs, which could garner significant interest. Additionally, Wang et al. discovered that the autophagy lysosome pathway (ALP), which is involved in the degradation of IGF2BP2, can be inhibited by *LINRIS* (Long Intergenic Noncoding RNA for IGF2BP2 Stability) [106]. This finding not only clarifies the degradation pathway of IGF2BP2 but also establishes a link between the lncRNA epigenetic network and m^6^A modification. However, it is important to note that, aside from the ALP, ubiquitinated cellular proteins are also degraded through the ubiquitin proteasome system (UPS) [107]. Given this, a pertinent question for future research is whether there exists a molecular mechanism that mediates the degradation of IGF2BP2 via the UPS, thereby potentially altering its expression levels. Answering this question could significantly expand our understanding of the regulatory pathways for IGF2BP2.

In summary, the action of lncRNAs such as LINC01232, circPACRGL, PACERR, circITGB6, and circASPH enables IGF2BP2 to enhance the stability of mRNAs for TGFBR1, YAP1, c-myc, and FGF9. This stabilization facilitates the polarization of M2 macrophages, potentially accelerating the onset and progression of NSCLC, OC, CRC, and pulmonary inflammation. However, the research has some limitations. M2 macrophages can be classified into three subgroups—M2a, M2b, and M2c—which exhibit distinct phenotypes and secreted substances, and thus have varying effects on diseases and tissue damage [108]. M2a macrophages are induced by IL-4 or IL-13, while M2b macrophages are activated by immune complexes, Toll-like receptors, or IL-1R agonists. Both M2a and M2b subgroups exert immunomodulatory functions and drive type II immune responses. In contrast, M2c macrophages, which are induced by IL-10, are more closely associated with immune suppression and tissue remodeling [108, 109]. Notably, it remains unclear which specific subgroups of M2 macrophages are regulated by IGF2BP2 during the process of promoting M2 macrophage polarization. Uncovering the answer to this question could be of great significance. It may enable us to gain a more profound understanding of the mechanism by which IGF2BP2 mediates tumor immune escape through the modulation of M2 macrophage polarization.

#### IGF2BP2 promotes inflammation by promoting polarization of M1 macrophages

M1 macrophages represent the primary phenotype in normal immune responses, engaging in TH1 (type I T helper cell) responses to various pathogens and producing pro-inflammatory cytokines that can eliminate tumor cells and inhibit microbial activity [110, 111]. M1 macrophages are classically activated by interferon-gamma (IFN-γ) or lipopolysaccharide (LPS), leading to the production of pro-inflammatory cytokines such as tumor necrosis factor-alpha (TNF-α), interleukin-6 (IL-6), and IL-1β. These cytokines facilitate microbial phagocytosis and initiate immune responses [112]. IGF2BP2 in tumor cells has also been identified as playing a crucial role in the polarization of M1 macrophages [113]. For instance, Lin et al. confirmed that circular RNA UBX domain protein 7 (CircUBXN7) promotes macrophage infiltration and renal fibrosis in diabetic nephropathy (DKD), a process related to IGF2BP2-mediated stabilization of the Sp1 transcription factor (SP1) mRNA. CircUBXN7 is significantly upregulated in the plasma of DKD patients. By binding directly to IGF2BP2, it enhances the stability and activation of SP1 mRNA, ultimately promoting M1 macrophage polarization, inhibiting M2 macrophage polarization, and increasing macrophage infiltration, EMT, fibrosis, and proteinuria in vivo [113].

Similarly, IGF2BP2 in macrophages has been found to promote M1 macrophage polarization [114, 115]. Ji et al. reported that, after miR-221-3p, which is carried by exosomes derived from breast epithelial cells, enters macrophages, this microRNA can promote the polarization of M1 macrophages by targeting IGF2BP2, playing a crucial role in the development of mastitis [114] (Fig. 4). Following treatment with LPS or LPS-derived exosomes (LPS-exo), miR-221-3p enhances macrophage migration and chemotaxis toward breast tissue by targeting IGF2BP2. However, the mechanism by which IGF2BP2 contributes to this phenotype remains to be elucidated, and the specific molecular mechanism by which miR-221-3p regulates IGF2BP2 expression is still unclear [114]. Further exploration is needed to address these issues. Additionally, Du et al. confirmed that, in THP-1-derived M0 macrophages, the METTL3/IGF2BP2 complex promotes M1 macrophage polarization by stabilizing the mRNA encoding T cell immunoglobulin mucin 1 (TIM1) [115]. When the polarization of macrophages is abnormal, it exacerbates inflammatory responses, thereby affecting the occurrence of sepsis and related organ damage [115].

In summary, the interaction between IGF2BP2 and circUBXN7 can enhance the stability of SP1 mRNA through m^6^A modification, or this interaction can be regulated by miR-221-3p. This enhancement subsequently promotes the polarization of M1 macrophages and accelerates the progression of DKD or mastitis.

### IGF2BP2 enhances the secretion of various pro-inflammatory cytokines in a non-macrophage-dependent manner, promoting inflammatory responses

#### IGF2BP2 stabilizes the mRNA of downstream target genes through m^6^A modification, enhances the secretion of various pro-inflammatory cytokines, and promotes inflammatory response

In recent years, numerous studies have demonstrated that IGF2BP2 can stabilize the mRNA of downstream target genes through m^6^A modification, leading to an increase in the secretion of various pro-inflammatory cytokines in a non-macrophage-dependent manner. This enhancement of inflammatory responses ultimately affects disease progression [116–119]. As a key enzyme in catalyzing m^6^A modification of RNA, METTL3 has been shown by Wang et al. to modulate programmed cell death and renal inflammation in tubular epithelial cells (TECs) stimulated by TNF-α, cisplatin (CDDP), and LPS. Silencing METTL3 alleviates these effects, while its overexpression has the opposite impact [116]. METTL3 promotes m^6^A modification of TGF-beta activated kinase 1 binding protein 3 (TAB3), increasing TAB3 mRNA stability by binding IGF2BP2 to its m^6^A-modified stop codon region. This process ultimately promotes the production of pro-inflammatory cytokines (TNF-α and IL-6), chemokines (MCP-1), and adhesion molecules (ICAM-1), accelerating kidney injury and inflammatory responses [116]. Jiang et al. discovered that METTL3 enhances the stability of TIMP metallopeptidase inhibitor 2 (TIMP2) mRNA in podocytes from kidney biopsies of DKD patients in an IGF2BP2-dependent manner. Subsequently, TIMP2 upregulates the expression of Notch3 and Notch4, increasing the secretion of pro-inflammatory factors such as TNF-α and IL-1β. This leads to pro-inflammatory and pro-apoptotic effects, accelerating podocyte injury in DKD [117]. Zhou et al. also revealed that IGF2BP2 enhances the stability of stromal interaction molecule 1 (STIM1) mRNA in LPS-induced A549 cells. Inhibition of STIM1 expression regulated by IGF2BP2 reduces LPS-induced endoplasmic reticulum stress (ERS) and inflammatory responses in A549 cells, mitigating cell damage in pneumonia cell models [118]. Furthermore, IGF2BP2 plays a significant role in the secretion of pro-inflammatory cytokines by tumor cells. Ji et al. found that the expression and m^6^A modification level of colony-stimulating factor 2 (CSF2) were significantly increased in gastric cancer mesenchymal stem cells (MSCs). Mechanistically, IGF2BP2 binds and stabilizes CSF2 mRNA in GC MSCs, inducing normal MSCs to reprogram into pro-cancer MSCs. This reprogramming enhances the proliferation, migration, and drug resistance of gastric cancer cells through the secretion of multiple pro-inflammatory factors [119].

In summary, IGF2BP2 plays a significant role in stabilizing the mRNA of TAB3, TIMP2, STIM1, and CSF2 through m^6^A modification. This stabilization enhances the secretion of pro-inflammatory cytokines, such as TNF-α, IL-1β, and IL-6, which in turn accelerates kidney injury, DKD podocyte injury, and cell damage in pneumonia cell models. It also promotes inflammatory responses or accelerates GC progression. However, there are limitations in the study by Zhou et al. They did not validate the role of IGF2BP2 in stabilizing STIM1 mRNA through in vitro experiments. Furthermore, while they found that inhibiting STIM1 expression regulated by IGF2BP2 can affect tumor cells, it remains unclear whether downregulation of IGF2BP2 indirectly affects STIM1 expression. These questions warrant further exploration in future experimental studies.

#### The upstream regulatory factors of IGF2BP2 affect its function, thereby enhancing the secretion of pro-inflammatory cytokines and exacerbating cellular inflammatory responses

Furthermore, IGF2BP2 can augment the secretion of IL-6 and MCP-1 by activating signaling pathways such as Toll-like receptor 4 (TLR4)/nuclear factor kappa-B (NF-κB), thereby exacerbating inflammation and damage to adipocytes and trophoblasts [120, 121]. For instance, You et al. demonstrated that, in mature 3T3-L1 adipocytes stimulated by TNF-α, lncRNA MGE3 can activate the TLR4/NF-κB signaling pathway by increasing the protein expression of IGF2BP2. This activation subsequently leads to elevated secretion levels of inflammatory factors IL-6 and MCP-1, enhanced cell apoptosis, and increased caspase-3 activity, ultimately worsening adipocyte inflammatory damage and insulin sensitivity imbalance [120]. Concurrently, Huang et al. discovered that circSESN2 can exacerbate high glucose (HG)-induced trophoblast damage by binding to IGF2BP2 and upregulating its protein expression. The inhibition of circSESN2 or IGF2BP2 not only promotes the invasion and migration of HTR-8/SVneo cells but also reduces cell apoptosis, pro-inflammatory cytokine release, and oxidative stress damage [121]. However, the precise mechanism by which IGF2BP2 influences the secretion of pro-inflammatory cytokines following upregulation by circSESN2 remains unclear, and additional experimental evidence is required to clarify this relationship. In summary, lncRNA MGE3 or circSESN2 can upregulate the expression of IGF2BP2, activating the TLR4/NF-κB signaling pathway or other related pathways, and enhancing the secretion of pro-inflammatory cytokines such as IL-6 and MCP-1. This leads to increased inflammatory damage to adipocytes and trophoblasts and accelerates cell apoptosis.

## IGF2BP2 regulates cellular lipid metabolism

The regulation of lipid metabolism, including lipid uptake, synthesis, and hydrolysis, is essential for maintaining cellular homeostasis [122]. Cancer cells within the tumor microenvironment can exploit lipid metabolism to fuel their rapid proliferation, survival, migration, invasion, and metastasis [123]. Numerous studies have indicated that m^6^A modification is associated with the progression of lipid metabolism disorders in humans [124, 125]. As the most abundant epigenetic modification of mRNA, m^6^A modification is crucial for regulating mRNA levels of genes related to lipid metabolism [126, 127]. Consequently, in recent years, researchers have observed that IGF2BP2 can enhance the stability or translation of mRNAs encoding lipid metabolism-related proteins such as platelet glycoprotein 4 (CD36), fatty acid binding protein 5 (FABP5), EBF transcription factor 2 (EBF2), and peroxisome proliferator-activated receptor γ (PPARγ). This enhancement affects lipid metabolism and accelerates the progression of nonalcoholic fatty liver disease (NAFLD), obesity, and obesity-related diseases.

### IGF2BP2 enhances the mRNA stability of downstream target genes through m^6^A modification, promoting lipid accumulation

**Fig. 5:**
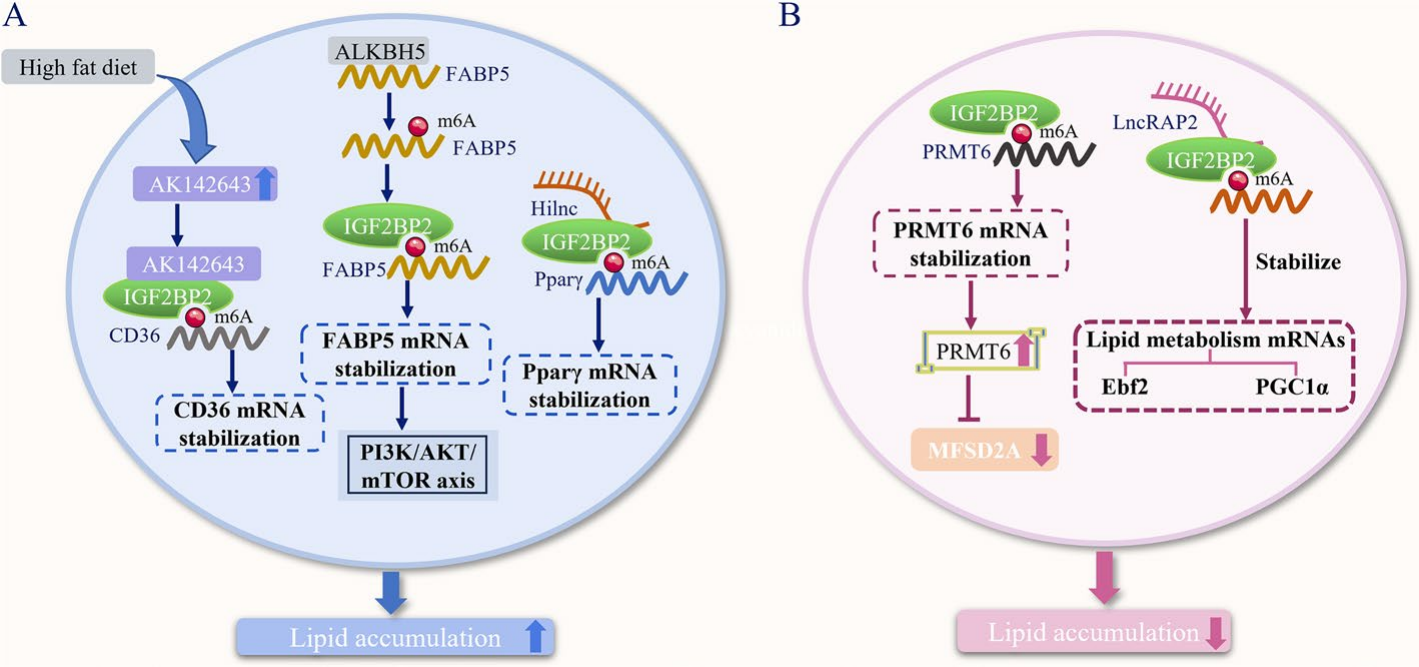
IGF2BP2 regulates cellular lipid metabolism. **A** IGF2BP2 can enhance the stability of CD36, PPARγ, and FABP5mRNA through m^6^A-modification-dependent mechanisms when interacting with AK142643 and Hilnc, as well as being influenced by ALKBH5, promoting lipid accumulation in liver and pancreatic neuroendocrine tumor cells. **B** IGF2BP2 can enhance the stability of thermogenic factors Ebf2, PPARγ, and PRMT6 mRNA in white adipocytes, ultimately reducing lipid accumulation. *CD36* platelet glycoprotein 4, *ALKBH5* human Alk B homolog 5, *FABP5* fatty acid binding protein 5, *PI3K/AKT/mTOR* phosphoinositide 3-kinase/protein kinase B/mammalian target of rapamycin, *PPARγ* peroxisome proliferator-activated receptor γ, *PRMT6* protein arginine N-methyltransferase 6, *MFSD2A* major facilitator superfamily domain-containing protein 2A, *Ebf2* EBF transcription factor 2, *PGC1α* peroxisome proliferator-activated receptor γ coactivator 1alpha. Microsoft PowerPoint was used to create this graphic

Over the past decades, the global prevalence of metabolic diseases such as steatohepatitis, type 2 diabetes, and myocardial infarction has risen significantly [128, 129]. These obesity-related diseases are partly attributed to the abnormal accumulation of harmful lipid metabolites in tissues not designed for lipid storage, such as the liver, vascular system, and pancreatic beta cells [129]. Research has confirmed that IGF2BP2-m^6^A modification promotes lipid accumulation by regulating the levels of proteins involved in lipid metabolism. For instance, Wang et al. demonstrated that AK142643 can interact with IGF2BP2 in the livers of mice fed a high-fat diet (HFD). This interaction subsequently enhances the stability of CD36 mRNA, leading to increased hepatic lipid accumulation both in vivo and in vitro, ultimately impacting hepatic lipid metabolism and accelerating the progression of NAFLD [130]. Furthermore, Jiang et al. revealed the pivotal role of the Hilnc-IGF2BP2 signaling axis in lipid metabolism. Hilnc can directly interact with IGF2BP2, enhancing the stability of PPARγ mRNA and thereby promoting diet-induced obesity and hepatic steatosis [131]. Additionally, Chen et al. uncovered a mechanism by which abnormal m^6^A modifications in the ALKBH5/IGF2BP2/FABP5/mTOR axis affect lipid metabolism, offering a new molecular basis for the development of therapeutic strategies for pancreatic neuroendocrine tumors (pNENs). Mechanistically, overexpression of ALKBH5 increases the stability of FABP5 mRNA in a manner dependent on m^6^A-IGF2BP2, thereby promoting fatty acid synthesis through the phosphoinositide 3-kinase/protein kinase B/mammalian target of rapamycin (PI3K/AKT/mTOR) signaling pathway [132] (Fig. 5).

In summary, IGF2BP2, by recognizing m^6^A modification sites, can enhance the stability of mRNAs encoding CD36, PPARγ, and FABP5, which promotes lipid accumulation in the liver and pancreatic neuroendocrine tumor cells. This enhancement accelerates the progression of NAFLD, obesity, hepatic steatosis, and pancreatic neuroendocrine tumors. In the study by Jiang et al., Hilnc was found to directly interact with IGF2BP2, thereby enhancing the stability of PPARγ mRNA. However, it remains unclear whether Hilnc, like AK142643, stabilizes PPARγ mRNA by facilitating the binding of IGF2BP2 to downstream target gene mRNA. The specific molecular mechanisms at play need further investigation to be fully understood.

### IGF2BP2 enhances the mRNA stability of downstream target genes through m^6^A modification, reducing lipid accumulation

Adipose cells are rich in mitochondria and play a crucial role in energy homeostasis by dissipating energy in the form of heat or through metabolic processes [133]. Alvarez Dominguez et al. demonstrated that the levels of lncRAP2 and IGF2BP2 in adipocytes decrease during the development of obesity and diabetes. IGF2BP2 not only promotes lipid accumulation but also regulates the stability or translation of downstream target gene mRNAs, which subsequently affects the energy consumption of adipocytes, promotes lipolysis, and reduces lipid accumulation. Mechanistically, in mature white adipocytes, lncRAP2 can form a complex with IGF2BP2. This lncRAP2–IGF2BP2 complex supports the energy expenditure of mature adipocytes by binding to and stabilizing numerous mRNAs encoding metabolic effectors, including EBF2 and peroxisome proliferator-activated receptor gamma coactivator 1-alpha (PGC1α) [134] (Fig. 5). However, it is not entirely clear whether lncRAP2, similar to Hilnc, regulates the affinity of IGF2BP2 for downstream target gene mRNAs. Further exploration is needed to elucidate this aspect of their interaction.

In addition to influencing the energy consumption of adipocytes, IGF2BP2 has also been found to affect the levels of docosahexaenoic acid (DHA), which is crucial for maintaining the function of human and mouse LSCs. IGF2BP2 stabilizes the mRNA of protein arginine methyltransferase 6 (PRMT6) through m^6^A modification, inhibiting the expression of the lipid transporter MFSD2A. This reduction in DHA levels helps maintain LSC function and ultimately promotes the development of AML [135] (Fig. 5). While this study suggests that the IGF2BP2–PRMT6–MFSD2A pathway influences DHA levels and plays a role in AML, the specific mechanisms underlying LSC-related fatty acid metabolism remain unclear. Furthermore, although IGF2BP2 can impact the metabolism of DHA and fatty acids in AML cells through the PRMT6–MFSD2A signaling axis, it is yet to be determined whether IGF2BP2 can directly regulate DHA levels.

In summary, IGF2BP2 plays a significant role in white adipocytes by enhancing the stability of thermogenic factor mRNAs, such as EBF2, PPARγ, and PRMT6, in an m^6^A-modification-dependent manner. This enhancement of mRNA stability ultimately leads to reduced lipid accumulation and decreased docosahexaenoic acid (DHA) levels, which in turn can influence the development of various diseases [134, 135].

## The relationship between IGF2BP2 and the prognosis or treatment of diseases

### IGF2BP2 is associated with poor prognosis in various diseases

Prognosis is a vital aspect of cancer diagnosis, influencing treatment decisions and the intensity of therapy [136]. Identifying prognostic biomarkers is crucial as it offers new prognostic insights and aids in pinpointing effective treatment targets. Studies indicate that IGF2BP2 is overexpressed in HNSCC tissues, playing a significant role in cellular processes such as synthesis, metabolism, growth, death, and motility [137]. Its association with lymph node metastasis and poor prognosis makes it a critical independent adverse prognostic factor in HNSCC [48, 138]. Similarly, in OSCC, elevated IGF2BP2 expression correlates with poorer overall survival, suggesting its potential as a prognostic biomarker [139, 140]. IGF2BP2 impacts on biological functions and pathways, such as EMT, glycolysis, cell cycle, and immune responses, and contributes to a poor prognosis in OSCC [141, 142]. In PDAC, IGF2BP2 upregulation is linked to poor prognosis and the immunosuppressive tumor microenvironment [143, 144]. In glioblastoma, IGF2BP2’s interaction with lncRNA CACS9 to enhance its stability suggests a poor prognosis for glioblastoma multiforme (GBM) [145]. This interaction stabilizes human kallikrein 2 (HK2) mRNA, and the disruption of this complex by CASC9 depletion affects IGF2BP2’s function. The reliance on CACS9 in GBM cells and the uniqueness of this complex warrant further investigation. The prognostic significance of IGF2BP2 extends to other cancers, including pancreatic cancer [66, 146], AML [67], HCC [147], LUAD [148], and CRC [149, 150], where its upregulation is associated with poor outcomes and serves as a potential diagnostic and therapeutic target. In summary, understanding the prognostic implications of IGF2BP2 is essential for disease diagnosis and may offer novel approaches to treatment.

### IGF2BP2 can promote chemoradiotherapy resistance in various diseases

Radiotherapy, which utilizes high-energy photon radiation such as X-rays and gamma rays, is a crucial method for treating cancer by directly and indirectly targeting cancer cells and tumor tissue to destroy them [151, 152]. Since its discovery by Nobel laureate Marie Curie, radiation has been recognized as a significant and effective tool in oncology for killing or controlling tumor growth [153]. Recently, Zhou et al. discovered that overexpression of IGF2BP2 in lung cancer cells can promote radiation resistance, while its inhibition can impair this resistance both in vitro and in vivo [154]. IGF2BP2 enhances the stability and translation of L-type amino acid transporter 1 (SLC7A5) mRNA through m^6^A modification, which in turn boosts SLC7A5-mediated methionine transport to produce *S*-adenosylmethionine. This leads to an increase in the trimethylation of histone H3 lysine 4 (H3K4me3), providing a positive feedback loop to the IGF2BP2 promoter region and upregulating its expression. This loop, through the AKT/mTOR pathway, enhances LUAD radiation resistance, potentially hindering the effectiveness of LUAD radiotherapy [154]. Additionally, research has indicated that circular RNA circEYA3 can decrease the radiosensitivity of HCC cells through the IGF2BP2/DTX3L axis. CircEYA3 binds to IGF2BP2, enhancing its ability to stabilize DTX3L mRNA, which specifically reduces radiation-induced DNA damage in HCC cells and increases their resistance to ^125^I radiation seeds and external irradiation.

Chemotherapy is a central treatment strategy for the majority of cancer patients, and drug resistance can lead to treatment failure, making the understanding and study of chemotherapy resistance a significant challenge in cancer biology [155]. Research has shown that IGF2BP2 can stabilize the mRNA of downstream target genes, such as dipeptidyl peptidase-4 (DPP4), DANCR, hydroxy-3-methylglutaryl-CoA synthase 1 (HMGCS1), and ATPase copper transporting alpha (ATP7A), in an m^6^A-modification-dependent manner in various tumor cells, including those from papillary thyroid carcinoma, glioblastoma, prostate, and OC. This stabilization enhances resistance to drugs such as cisplatin, selumetinib, etoposide, docetaxel, and temozolomide, ultimately promoting disease progression [156–163]. Furthermore, evidence suggests that, in glioblastoma and cervical cancer cells, lncRNAs OIP5-AS1 and miR-96-5p upregulate IGF2BP2 expression through interactions with microRNA-129-5p and lncRNA TRIM52-AS1, respectively. This upregulation promotes tumor cell resistance to temozolomide and cisplatin, inhibits tumor cell apoptosis, and accelerates tumor progression [164, 165].

In summary, IGF2BP2 plays a significant role in tumor radiotherapy and chemotherapy, with its impact mediated through m^6^A-modification-dependent mechanisms. Recent findings have highlighted the potential of IGF2BP2 small-molecule inhibitors in treating certain cancers, such as T-cell acute lymphoblastic leukemia and colorectal cancer [161, 166]. However, for many tumor types, including lung cancer, small-molecule inhibitors targeting IGF2BP2 have yet to be discovered. Consequently, the development of IGF2BP2 small-molecule inhibitors as targeted therapies for a range of diseases is likely to garner considerable interest from the research community.

## Conclusions

As a well-studied m^6^A reader, IGF2BP2 has been confirmed by numerous studies to participate in a variety of biological and pathogenic processes. It achieves this by regulating the stability of downstream target gene mRNAs and is intimately linked to the onset and progression of cancer. The key roles played by ferroptosis, EMT, cell stemness, angiogenesis, inflammatory response, and lipid metabolism in physiological and pathological processes such as tumors, immunity, and development have always been the focus of researchers’ attention. Understanding the regulatory interplay between IGF2BP2 and these processes, along with the specific molecular mechanisms involved, is anticipated to yield valuable new strategies for targeting IGF2BP2 in cancer treatment and drug development. Currently, research on IGF2BP2 predominantly focuses on its role in regulating mRNA stability through m^6^A modification. In contrast, there is a relative dearth of research on other molecular mechanisms that IGF2BP2 might employ. Next, a deeper understanding of the specific molecular mechanisms by which IGF2BP2 regulates downstream target gene expression may improve this situation, and may provide fresh insights and potentially therapeutic approaches.

## Outstanding questions

- 1. In addition to regulating macrophage polarization, can IGF2BP2 also influence the differentiation of other immune cells associated with tumors, thereby exerting a more profound effect on the tumor microenvironment?
- 2. In the same RNA molecule, there are typically multiple N^6^-methyladenosine (m^6^A) modification sites and several m^6^A readers. Is there a specific sequence that m^6^A readers recognize at m^6^A sites? Is there a competitive relationship between other m^6^A readers and IGF2BP2?
- 3. Besides recruiting EIF4A1 to enhance the translation rate of CDK6, can IGF2BP2 also upregulate the translation rates of other downstream target genes? Furthermore, does IGF2BP2 possess other regulatory mechanisms that affect the expression of these downstream target genes?

## Data Availability

Not applicable.

## Abbreviations

IGF2BP2: Insulin like growth factor 2 messenger RNA binding protein 2
snRNA: Small nuclear RNA
APA: Alternative polyadenylation
FTO: Obesity-associated protein
HuR: ELAV-like RNA-binding protein 1
MATR3: Matrin 3
m^6^A: N^6^-methyladenosine
EMT: Epithelial–mesenchymal transition
IGF2BPs: Insulin-like growth factor 2-messenger RNA binding proteins family
RBP: RNA-binding proteins
KH: K-homology
LncRNA: Long noncoding RNA
GSH: Glutathione
GPX4: Glutathione peroxidase 4
PTC: Papillary thyroid cancer
ESCC: Esophageal squamous cell carcinoma
HPSCC: Hypopharyngeal squamous cell carcinoma
SLC7A11: Solute carrier family 7, membrane 11
HIF-1: Hypoxia-inducible factor
ATF3: Activating transcription factor 3
ROS: Reactive oxygen species
LPO: Lipid peroxidation
UC: Ulcerative colitis
DSS: Dextran sulfate sodium
MDA: Malondialdehyde
TMEM44-AS1: TMEM44 antisense RNA 1
ALKBH5: AlkB homolog 5
NRF2: Nuclear factor erythroid 2-related factor 2
SAT1: Spermidine/spermine N1-acetyltransferase 1
ACSL4: Acyl-CoA synthetase long-chain family 4
PMNs: Polymorphonuclear neutrophils
NETs: Neutrophil extracellular traps
SI-ALI: Sepsis-induced acute lung injury
METTL3: Methyltransferase-like 3
SAP: Severe acute pancreatitis
PMVECs: Primary microvascular endothelial cells
circEXOC5: Circular RNA exocyst complex component 5
TNBC: Triple-negative breast cancer
CDK6: Cyclin-dependent kinase 6
TFs: Transcription factors
SNAI1/2: Snail family transcriptional repressor 1/2
TWIST1/2: Twist family bHLH transcription factor 1/2
ZEB1/2: Zinc finger E-box binding homeobox 1/2
TIM1: Mucin 1
HMGA1: High mobility group A1
HNSCC: Head and neck squamous cell carcinoma
GC: Gastric cancer
CDS: Coding sequence
DHX9: DExH-box helicase 9
LINC00460: Long intergenic non-protein-coding RNA 460
CRC: Colorectal cancer
UTR: Untranslated region
CDC27: Cell division cycle 27
LINC01559: Long intergenic non-protein-coding RNA 1559
HCC: Hepatocellular carcinoma
TCF7L2: Transcription factor 7-like 2
PC: Pancreatic cancer
MAFG-AS1: MAF BZIP transcription factor G antisense RNA 1
MSC-AS1: Musculin antisense RNA 1
ETV1: ETS translocation variant 1
LEF1: Lymphoid enhancer binding factor 1
Src: Nonreceptor tyrosine kinase gene
FAK: Focal adhesion kinase
MEK: Mitogen-activated protein kinase kinase
EREG: Epiregulin
OSCC: Oral squamous cell carcinoma
CSCs: Cancer stem cells
DANCR: Differentiation antagonizing non-protein-coding RNA
LSCs/LIC: Leukemia stem cells/initiating cells
Gln: Glutamine
Glu: Glutamate
eIF: Eukaryotic translation initiation factor
MYC: V-Myc avian myelocytomatosis viral oncogene homolog
SLC1A5: Solute carrier family 1 member 5
GPT2: Glutamic–pyruvic transaminase 2
AML: Acute myeloid leukemia
SOX2: Sex determining region Y-Box 2
PCa: Prostatic carcinoma
CCAR1: Cell division cycle and apoptosis regulator 1
CDC45: Cell division cycle 45
OCT4: Octamer-binding transcription factor 4
HSCs: Hematopoietic stem cells
OIP5-AS1: Opa interacting protein 5 antisense RNA 1
miR-495-3p: MicroRNA-495-3p
VM: Vasculogenic mimicry
LUAD: Lung adenocarcinoma
FLT4: Fms-related receptor tyrosine kinase 4
PI3K: Phosphatidylinositol-3-kinase
Akt: Protein kinase B
Lnc01166: Long intergenic non-protein-coding RNA 1116
AAMP: Angio-associated migratory cell protein
miR-210-3p: MicroRNA-210-3p
HPSE: Heparinase
NRARP: NOTCH regulated ankyrin repeat protein
ESM1: Endothelial cell specific molecule 1
BCa: Bladder cancer
AGAP2-AS1: AGAP2 antisense RNA 1
LRG1: Leucine-rich α-2 glycoprotein 1
ZNF281: Zinc finger protein 281
LncSNHG5: Long noncoding RNA small nucleolar RNA host gene 5
CCL2: C–C motif chemokine ligand 2
CCL5: C–C motif chemokine ligand 5
P38 MAPK: P38 mitogen-activated protein kinase
circMET: Circular RNA MET
TAI: Tumor-associated inflammation
LAUD: Lung adenocarcinoma
TAMs: Tumor-associated macrophage
NSCLC: Non-small cell lung cancer
THP-1: Human monocytic-leukemia cells
circPACRGL: Circular RNA Parkin coregulated like
YAP1: Yes1 associated transcriptional regulator
circITGB6: Circular RNA integrin subunit beta 6
circASPH: Circular RNA aspartate beta-hydroxylase
OC: Ovarian cancer
FGF9: Fibroblast growth factor 9
STING: Stimulator of interferon genes
CDDP: Cisplatin
PACERR: PTGS2 antisense NFKB1 complex-mediated expression regulator RNA
LINC01232: Long intergenic non-protein coding RNA 1232
KLF12: Kruppel-like factor 12
c-myc: Transcriptional regulator Myc-like
TGFBR1: Transforming growth factor beta receptor 1
PDAC: Pancreatic ductal adencarcinoma
TSC1: Tuberous sclerosis complex 1
PPAR γ: Peroxisome proliferator-activated receptor γ
CRE: Cockroach allergen
mTORC1: Mechanistic target of rapamycin complex 1
ALP: Autophagy lysosome pathway
UPS: Ubiquitin proteasome system
TH1: Type I T helper cells
IFN – γ: Interferon-gamma
LPS: Lipopolysaccharide
TNF-α: Tumor necrosis factor-α
IL-6: Interleukin-6
DKD: Diabetes nephropathy
SP1: Sp1 transcription factor
LTA: Lipoteichoic acid
TECs: Tubular epithelial cells
TAB3: TGF-beta activated kinase 1 binding protein 3
MCP-1: Monocyte chemoattractant protein-1
ICAM-1: Intercellular adhesion molecule 1
TIMP2: TIMP metallopeptidase inhibitor 2
STIM1: Stromal interaction molecule 1
ERS: Endoplasmic reticulum stress
CSF2: Colony-stimulating factor 2
MSCs: Mesenchymalstem cells
TLR4: Toll-like receptor 4
NF -κB: Nuclear factor kappa-B
HG: High glucose
CD36: Platelet glycoprotein 4
FABP5: Fatty acid binding protein 5
EBF2: EBF transcription factor 2
NAFLD: Nonalcoholic fatty liver disease
HFD: High-fat diet
pNENs: Pancreatic neuroendocrine tumors
PI3K/AKT/mTOR: Phosphoinositide 3-kinase/protein kinase B/mammalian target of rapamycin
PGC1α: PPAR gamma coactivator factor 1 alpha
DHA: Docosahexaenoic acid
PRMT6: Protein arginine methyltransferase 6
GBM: Glioblastoma multiforme
HK2: Human kallikrein 2
SLC7A5: L-type amino acid transporter 1
H3K4me3: Histone H3 lysine 4
DPP4: Dipeptidyl peptidase-4
HMGCS1: Hydroxy-3-methylglutaryl-CoA synthase 1
ATP7A: ATPase copper transporting alpha
